# From the Gut to the Brain: Journey and Pathophysiological Effects of the Food-Associated Trichothecene Mycotoxin Deoxynivalenol

**DOI:** 10.3390/toxins5040784

**Published:** 2013-04-23

**Authors:** Marc Maresca

**Affiliations:** Aix Marseille Université, CNRS, iSm2 UMR 7313, Marseille 13397, France; E-Mail: m.maresca@univ-amu.fr; Tel.: +33-491-288-445; Fax: +33-491-284-440

**Keywords:** deoxynivalenol, mycotoxin, trichothecene, detoxification, intestinal absorption, intestine, brain, endocrine, glial cells, immune cells

## Abstract

Mycotoxins are fungal secondary metabolites contaminating food and causing toxicity to animals and humans. Among the various mycotoxins found in crops used for food and feed production, the trichothecene toxin deoxynivalenol (DON or vomitoxin) is one of the most prevalent and hazardous. In addition to native toxins, food also contains a large amount of plant and fungal derivatives of DON, including acetyl-DON (3 and 15ADON), glucoside-DON (D3G), and potentially animal derivatives such as glucuronide metabolites (D3 and D15GA) present in animal tissues (e.g., blood, muscle and liver tissue). The present review summarizes previous and very recent experimental data collected *in vivo* and *in vitro* regarding the transport, detoxification/metabolism and physiological impact of DON and its derivatives on intestinal, immune, endocrine and neurologic functions during their journey from the gut to the brain.

## 1. Introduction

**Figure 1:**
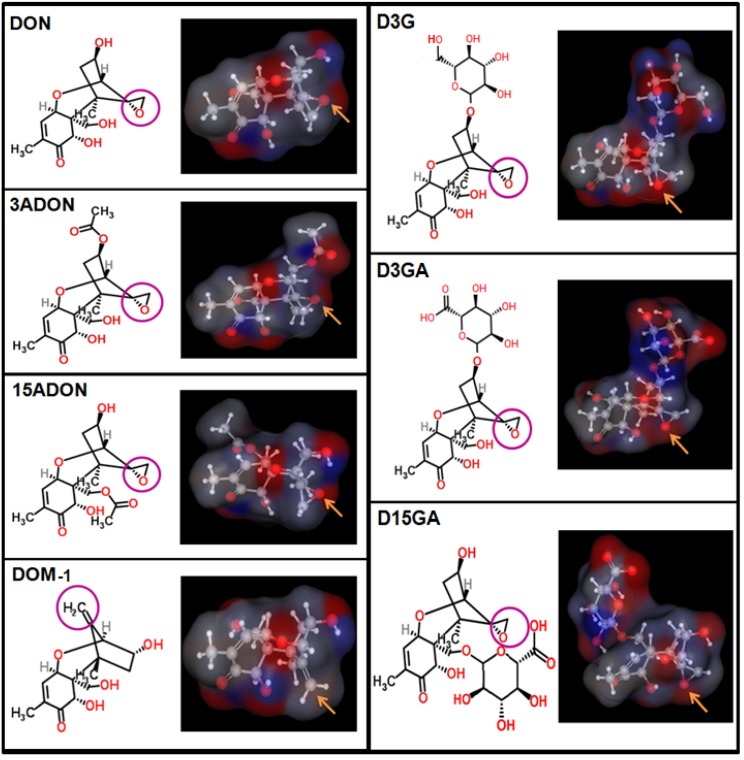
Chemical structure of DON and its major derivatives. DON and its derivatives were drawn using Marvin software. Images on the right show an electrostatic map of the molecules, the blue color indicating positive region, the red color indicating negative region and the gray color indicating neutral region. The purple circles on the left images and yellow arrows on the right images indicate the position of the epoxide or de-epoxide function in DON and its derivatives.

Deoxynivalenol (DON, vomitoxin) belongs to a family of mycotoxins called trichothecenes. Trichothecenes (including T-2 toxin, nivalenol, DON and satratoxins) are structurally related molecules produced by fungi of *Fusarium* and *Stachybotrys* species [1]. They are small sesquiterpenoids all having in common an epoxide group at position 12–13 that is critical for their toxicity [2,3,4,5] (Figure 1). It has been proposed that the epoxide group allows them to bind to ribosomes, a mechanism known as the ribotoxic stress effect, leading to the activation of various protein kinases, the modulation of gene expression, the inhibition of protein synthesis and cell toxicity [5,6,7,8].

Analyses of the occurrence of DON in food and feed matrices have demonstrated that DON is one of the most prevalent food-associated mycotoxins, particularly in cereals and cereal-derived products [9]. In the US, 73% and 92% of wheat and corn samples, respectively, were found positive for DON [10]. In Europe, a large-scale collaborative study conducted on more than 40,000 food samples has shown that DON was present in 57% of all samples, with a percentage of positive samples varying depending of the country (*i.e.*, from 15% to 100% for Belgium and France, respectively) and at levels ranging from 91 to 5000 µg/kg [11]. Similarly, another study conducted on 82 feed matrices in Europe has demonstrated that 67 of them were contaminated with DON, 52 samples being highly contaminated with levels of DON ranging from 74 to 9528 μg/kg [12]. DON is, moreover, resistant to high temperature (up to 350 °C), thereby making it stable during processing and cooking, leading to its persistence throughout the food chain [13].

In addition to its prevalence, DON is one of the most hazardous food-associated mycotoxins [4,7,8,13,14,15,16]. A provisional maximum tolerable daily intake (PMTDI) for DON of 1 µg/kg of body weight and per day has been proposed by the Joint FAO/WHO Expert Committee on Food Additives (JECFA) [14]. The ingestion of DON has been associated with alterations of the intestinal, immune and nervous systems, thus leading, in cases of acute exposure, to illnesses characterized by vomiting, anorexia, abdominal pain, diarrhea, malnutrition, headache and dizziness [4,7,8,17]. Toxicity of DON relies on its ability to cross the biological barriers (*i.e*., the intestinal and blood-brain barriers) and to affect the functions and viability of the cells forming such organ systems.

The present paper compiles experimental data collected *in vivo* and *in vitro* regarding: (i) the transport of DON and DON derivatives from the gut to the brain; (ii) their detoxification; and (iii) their impact on the animal and human physiology.

## 2. Transport and Metabolism of DON

### 2.1. Structure and Physicochemical Properties of DON and Its Derivatives

**Figure 2:**
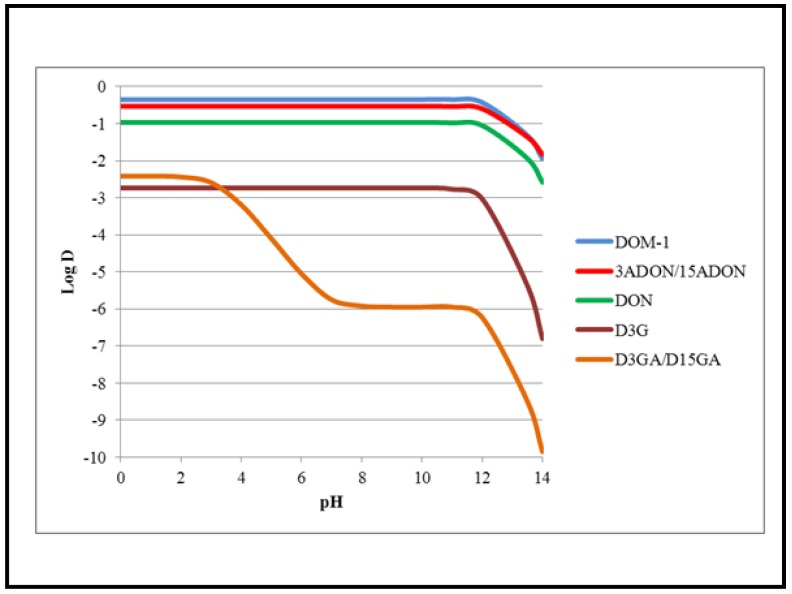
Log*D* values of DON and its derivatives. Log*D* values of DON and its derivatives at various pH values were calculated using Marvin software.

Food and feed are contaminated both by native DON and its derivatives. The structure and partition coefficient (log*D*) of DON and its metabolites are given in Figure 1, Figure 2. The major derivatives of DON correspond to metabolites formed either by fungi (*i.e*., the acetylated derivatives: 3- and 15-acetyl-DON or 3ADON and 15ADON), plants (*i.e*., 3-*O*-glucoside-DON or D3G), animals (*i.e*., glucuronic acid derivatives: DON-3 and DON-15-glucuronide or D3GA and D15GA) or bacteria (*i.e*., the de-epoxide diene derivatives of DON: DOM-1) [18,19]. Various studies have shown that food contains large amounts of DON metabolites, mainly the fungal and plant derivatives 3/15ADON and D3G, with up to 75% of the total amount of DON corresponding to DON metabolites [19]. In addition, although no studies confirm it, animal derivatives of DON (*i.e*., D3/15GA) may be theoretically present in food originated from animal tissues and blood. The amount of DON metabolites has not been considered in the regulatory limits fixed by food agencies for DON due to the lack of data regarding their absorption and toxicity [19].

Calculation of the partition coefficient demonstrates that metabolic modifications of DON lead to important changes in the polarity of the molecule (Figure 2). Log*D* of DON is −0.97 at pH 7, suggesting a polar behavior. The presence of an acetyl moiety in the fungal metabolites 3ADON and 15ADON or the absence of the oxygen linked to the epoxide function in the bacterial diene metabolite DOM-1, result in a decrease in the polarity of the molecule compared to the native toxin (log*D* values of the metabolites being less negative than the one of DON with a value at neutral pH of −0.35 and −0.53 for DOM-1 and 3/15ADON, respectively).

Conversely, the presence of a glucoside or a glucuronide moiety in the plant and animal metabolites D3G, D3GA and D15GA leads to an increase in their polarity compared to DON (their log*D* values being more negative than the one of the native toxin with a value at neutral pH of −2.74 and −5.75 for D3G and D3/15GA, respectively). As discussed below, increase or decrease in the polarity of DON metabolites may affect their ability to enter the cells and thus to be absorbed by the intestine and/or to cause cell toxicity.

### 2.2. Cell Entry of DON and Its Derivatives

**Figure 3:**
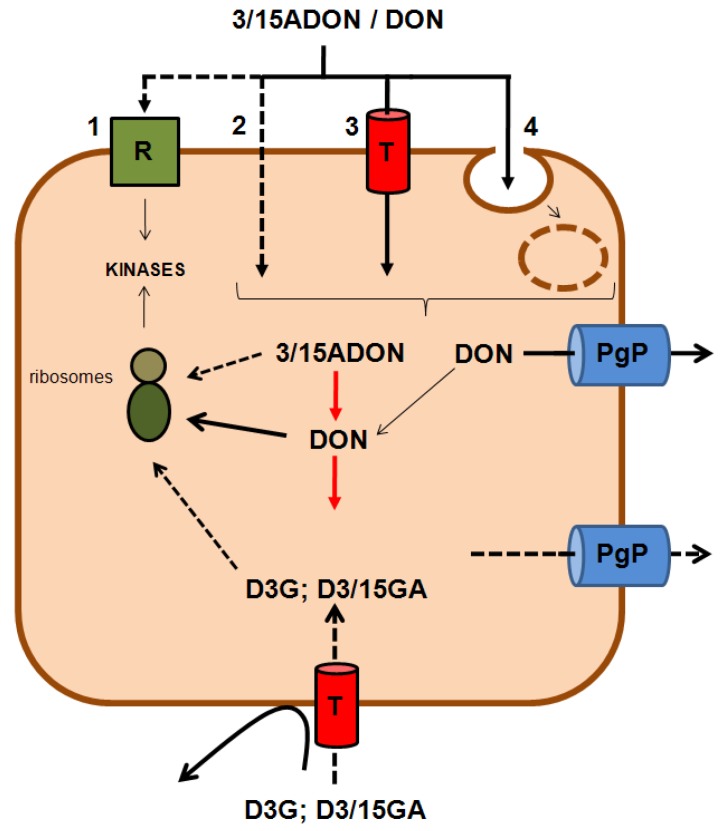
Cell entry of DON and its derivatives. Although highly unlikely, cellular effects of DON could rely on its ability to directly bind membrane receptor(s) (R) (**1**). However, the fact that DON interacts with ribosomes and is substrate of intracellular detoxification enzymes rather suggests that DON enters the cells. Cell entry of DON and its acetylated derivatives (3/15ADON) could take place through membrane diffusion across lipids (**2**), through a membrane transporter (T) (**3**) or through bulk phase endocytosis/pinocytosis (**4**) Once in the cell, 3/15ADON could be transformed in DON by intracellular carboxyl-esterases. DON (and possibly 3/15ADON) reacts then with ribosomes to cause cell effects. Detoxification of DON involves the production of glucuronide-metabolites by UDP-glucuronosyltransferases. In addition, P-glycoproteins (PgP) are responsible for the efflux/excretion of DON and possibly of its derivatives. The absence of cell effects of D3G and D3/15GA suggests either that: (i) these derivatives do not cross the cell membrane (**5**); or (ii) that they efficiently enter the cell but do not bind to ribosomes (**6**), the first hypothesis being more likely. Dashed lines/arrows and full lines/arrows indicate unlikely/hypothetical and likely mechanisms, respectively.

No studies have been conducted to characterize the exact mechanism of the cell entry of DON, with only speculations being possible at present (Figure 3). One possibility is that cell entry of DON does not occur at all and that cellular effects of DON described in part 3.1, such as activations of various kinases, rely on its interaction with membrane receptors/proteins activating such signal pathways. Although the direct effect of DON on membrane proteins could not be ruled out, data support the idea that at least a part of DON enters the cells, *i.e*., the fact that: (i) DON binds to intracellular ribosomes; and (ii) DON is substrate of intracellular detoxification enzymes (see part 2.4.). Studies using intestinal cells have shown that the cell entry of DON does not saturate when the extracellular concentration of toxin increases, suggesting that its entry takes place through a passive diffusion mechanism [20,21]. An important question is how DON, with its log*D* value of −0.97 at neutral pH that makes it behave like a polar molecule, could diffuse across the cell membrane. Based on the fact that only molecules bearing a log*D* value close to zero or positive are able to enter the cells through lipid diffusion [22], the ability of DON to enter the cells through such a mechanism is theoretically low to nil. This leaves only two possibilities for DON to cross the cell membrane: (i) a diffusion through an uncharacterized membrane-associated passive transporter; and/or (ii) a bulk phase endocytosis/pinocytosis process (Figure 3) [23,24].

As for DON, no data exists regarding the mechanism of cell entry of DON derivatives. As explained in Section 3.1., alterations in their ability to enter the cells and/or to bind to ribosomes/receptors may explain the difference of cell toxicity and toxicokinetics observed for DON derivatives compared to the native toxin. One can suppose that DON derivatives with log*D* values closer to zero (*i.e*., DOM-1 and 3/15ADON) may have higher ability to diffuse across the lipids of the membrane. Conversely, glucoside and glucuronide metabolites of DON (D3G, D3/15GA) bearing bigger molecular masses and more polarity would have a reduced ability to enter the cells through lipid diffusion. Similarly, modifications of DON (size, polarity) may also theoretically affect the ability of DON derivatives to interact with membrane transporters if such transporters are involved. Future studies should help identify the mechanism(s) that permit the entry of DON and its derivatives into the cells.

### 2.3. Bacterial Transformation and Intestinal Absorption of DON and Its Derivatives

Metabolism of DON in plants and fungi has been fully described recently [18,19] and thus will not be elaborated upon in the present review focusing on modifications of DON by bacteria, animals and humans.

The first phase of the intoxication by DON and its derivatives corresponds to their passage through the gut wall, such transport being possibly affected by bacterial metabolism. The intestinal tract of animals and humans contains vast amounts of bacteria forming the commensal microbiota that lives in symbiosis with the host. At present, the microbiota could be considered as an additional organ system, playing important roles in the maturation of the intestinal and immune systems, in the nutrition of the host, and finally in the protection of the host against pathogenic micro-organisms and hazardous chemicals/xenobiotics, including DON and its derivatives [18,25,26,27].

**Figure 4:**
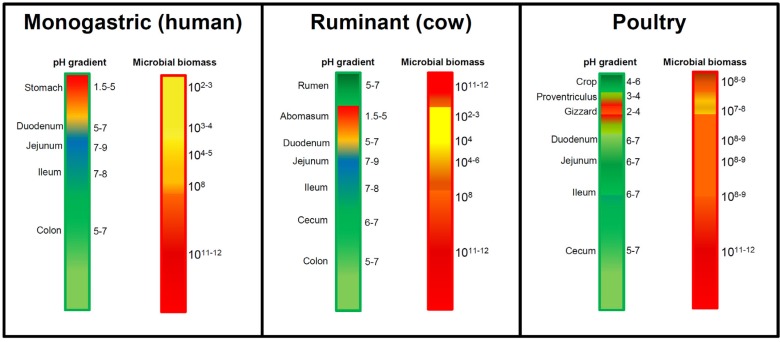
Regional pH and bacterial densities in the digestive tract. pH and bacterial density (per mL of intestinal fluid content) of the different segments of the digestive tract of humans, ruminants and poultry are indicated in the figure. Values were obtained from publications [27,28,29,30,31].

**Figure 5:**
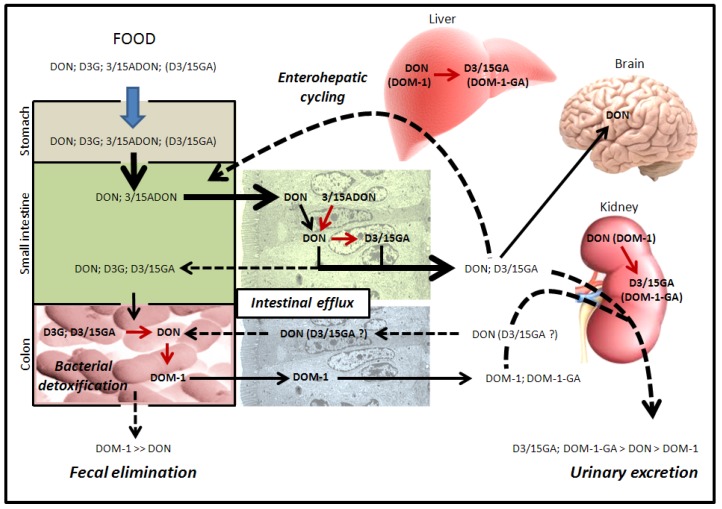
Intestinal absorption, detoxification and excretion of DON and its derivatives in monogastric species (e.g., humans/pigs/rodents). Humans and monogastric animals are exposed to DON and DON derivatives through the ingestion of contaminated food. Details are given in the text (parts 2.3 and 2.4). DOM-1-GA corresponds to glucuronide derivatives of DOM-1. Red arrows indicate transformation of DON or DON derivatives, dashed arrows indicate excretion/elimination mechanisms.

**Figure 6:**
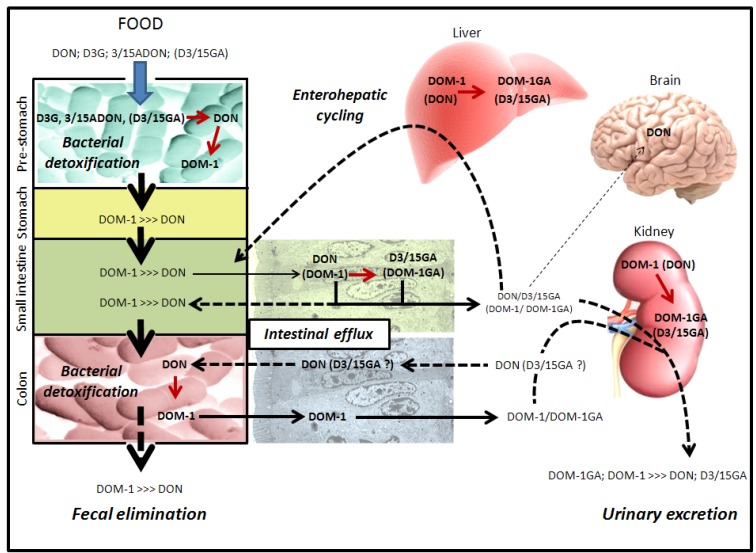
Intestinal absorption, detoxification and excretion of DON and its derivatives in ruminants and poultry. Poultry and polygastric animals are exposed to DON and DON derivatives through the ingestion of contaminated food. Details are given in the text (parts 2.3. and 2.4.). DOM-1-GA corresponds to glucuronide derivatives of DOM-1. Red arrows indicate transformation of DON or DON derivatives, dashed arrows indicate excretion/elimination mechanisms.

The efficiency of the intestinal absorption and metabolism of DON greatly varies between animal species due in part to the localization of bacteria along their intestine in relation to regional pH (Figure 4) [27,28,29,30,31]. On this basis, animals could be divided into two groups: (i) those with a high bacterial content located both before and after the small intestine such as polygastric animals (*i.e*., ruminants that have bacteria in their rumen and in their colon) and birds (including poultry that have bacteria in their crop and in their cecum); and (ii) those with high bacterial content located only after the small intestine, in their colon, such as most of the monogastric species (including humans, pigs and rodents). Localization of the gut bacteria prior or after the small intestine has a major effect on the bioavailability of ingested DON and its metabolites (Figure 5, Figure 6).

In monogastric animals, large amounts of ingested DON can cross the intestinal epithelium and reach the blood compartment (Figure 5). For example, in pigs, 54% to 89% of the ingested toxin is absorbed *in vivo* after acute and chronic oral exposure to DON, respectively [32], possible explanations for the higher oral bioavailability of DON after chronic exposure are discussed in the following. After oral intoxication of pigs, DON starts to appear in the plasma after 30 min and its serum concentration reaches a peak value within three to four hours post-ingestion, thereby suggesting a fast and efficient absorption of the toxin through the proximal small intestine [32,33,34]. Accordingly, *in vitro* experiments conducted with intestinal segments from pigs have shown that the intestinal absorption of DON takes place mainly through the jejunum [35]. Similarities between the human and pig intestines (also in terms of DON effects as described in part 3.2. suggest that humans could also efficiently absorb ingested DON.

Even though *in vivo* toxicokinetic data of DON in humans are not available, the intestinal absorption of DON by humans has been elegantly evaluated using an *in vitro* model of human intestinal epithelial cells (IEC), *i.e*., the Caco-2 cell line [20]. Caco-2 cells have been used for decades to mimic the human IEC and to study the intestinal absorption of drugs and toxins, this cell line giving apparent permeability coefficient (*P*app) values predictive of the *in vivo* oral bioavailability of molecules in humans [36]. Authors showed that DON has a *P*app value of 5.02 × 10^−6^ cm/s in Caco-2, corresponding to a potential *in vivo* oral bioavailability of 50% to 60% in human, a value in accordance with the *in vivo* experiments conducted on pigs. The *P*app of DON was also measured *in vitro* in poultry, giving even higher values (*i.e*., 1.7 × 10^−5^ cm/s, corresponding to a predicted *in vivo* oral bioavailability of 70%) [37]. As discussed below, the observed oral bioavailability of DON in poultry is much lower (*i.e*., around 20%) than expected, in relation to the presence of intestinal bacteria able to transform DON in DOM-1 before the small intestine in birds. Few data are available regarding the mechanism of intestinal absorption of DON. Experiments conducted *in vitro* with Caco-2 cells [20] or with intestinal segments from poultry [37] showed that the intestinal absorption of DON does not saturate but is proportionally dependent on the extracellular concentration of DON, thus suggesting that the intestinal absorption of DON takes place through passive transcellular and/or paracellular diffusion. As explained in part 2.2., based on its ability to target intracellular ribosomes and to be substrate of intracellular detoxification enzymes, at least a part of the intestinal absorption of DON has to take place through transcellular transport. Although the relative contribution of transcellular and paracellular transport in DON absorption has not been evaluated yet, contribution of the paracellular mechanism may massively increase and become predominant in the case of alterations of intestinal permeability. Decreases in the intestinal tightness are observed in various conditions such as inflammatory bowel disease (IBD) (including Crohn’s disease), intestinal infections by viruses, or pathogenic bacteria and exposure to DON or other mycotoxins [14,38,39,40,41,42,43,44]. This could explain the observed higher absorption of DON in pigs chronically exposed to oral DON compared to acute exposure [32].

The rank order of sensitivity of animals to ingested DON is pigs > poultry/ruminants [4]. As mentioned above, intestinal explants from poultry and pigs possess a similar ability to intestinally absorb DON, suggesting that the difference in their sensitivity to ingested DON does not rely on their ability to intestinally absorb DON. In fact, the sensitivity of animals to oral DON relies on the localization of the intestinal bacteria in their gut in relation to their ability to generate 9,12-diene DON or DOM-1, the non-toxic de-epoxide derivative of DON [18,45].

The presence of high bacterial contents that are able to convert toxic DON into its non-toxic de-epoxide metabolite DOM-1 before the small intestine in ruminants (rumen-associated bacteria) and poultry (crop-associated bacteria) massively decreases the amount of native DON reaching the small intestine, making such animal species almost insensitive to oral intoxication by DON (Figure 6) [4]. For example, only a small amount of the ingested DON reaches the small intestine as native toxin in poultry and sheep (19.3% and 7.5% of the ingested DON being found in the blood of intoxicated poultry and sheep, respectively) [46,47,48]. Similarly, in cows, 16% of ingested toxin reaches the small intestine [49] and only 1% crosses the intestinal wall to reach the blood [50].

In monogastric animals, due to the high absorption of DON by the small intestine, bacterial transformation of DON in DOM-1 could only be possible if a part of the ingested DON reaches the colon and/or in the case of intestinal/hepatic excretion of absorbed DON (Figure 5). This explains why only a low percentage of ingested DON is found in the feces of monogastric animals as DOM-1, with most of the ingested DON being eliminated in the urine as glucuronide-DON, DON, glucuronide-DOM-1 and DOM-1 (Figure 5) [51,52].

No studies have looked at the intestinal absorption of DOM-1 in animals or humans. However, based on the fact that DOM-1 is only formed by intestinal bacteria in the gut lumen and that a percentage of ingested DON is found in urine as DOM-1, we could suppose that DOM-1 formed by intestinal bacteria is efficiently absorbed by the gut (Figure 5, Figure 6) [53].

Not all bacteria are able to transform DON in DOM-1 [18]. In pigs, it has been demonstrated that only chronic oral exposure to DON leads to the formation of DOM-1 by the microbiota [54]. Experiments conducted with human feces coming from five volunteers showed that only one spontaneously possesses bacteria able to transform DON in DOM-1 [53]. Taken together, experiments conducted with pigs and humans suggest that naive intestinal bacteria naturally do not possess the ability to detoxify DON and that pre-exposure of the microbiota to DON induces the appearance of the bacterial detoxification activity, either through the induction of the expression of particular enzymes and/or the selection of particular detoxifying bacterial species [18]. Initially, aerobic bacteria were thought to be unable to form DOM-1 as they rather transform DON in 3-epi-DON and 3-keto-4-DON, both having an intact epoxide function [18,55]. However, recent data suggested that some soil bacteria are also able to form DOM-1 both in aerobic or anaerobic condition [56]. It has to be noted that although some bacteria and micro-organisms were initially thought to be able to totally mineralize DON, data suggest that adsorption of the toxin to the cell wall and bacterial uptake are in fact responsible for the disappearance of the toxin from the medium [18], with such adsorption certainly playing an important role in the neutralization of DON by the intestinal bacteria.

Fungal (*i.e*., 3/15ADON) and plant (*i.e*., D3G) metabolites of DON are also present in food and could thus be absorbed by the intestine and/or metabolized by intestinal bacteria (Figure 5, Figure 6). In addition, although no published studies describe it, animal derivatives of DON (*i.e*., D3/15GA) could also be theoretically present in animal-derived food (animal tissues, blood) and thus be ingested by humans or animals. In pigs, the ingestion of 3ADON leads to the appearance of DON (58%) and DON metabolites (glucuronide-DON and DOM-1 (42%)) but not of 3ADON in the blood [52]. This result suggests either that: (i) 3ADON is not directly absorbed by IEC but requires its initial transformation into DON by gut bacteria or by luminal intestinal lipases before its absorption; or (ii) 3ADON is directly absorbed by IEC that transform it intracellularly into DON before its release in the blood. Luminal intestinal lipases and microbial esterases/lipases could theoretically cut the acetyl moieties of the fungal metabolites 3ADON and 15ADON to release DON in the intestinal lumen [57,58]. Similarly, IEC possess intracellular carboxylesterases (CES) [59] potentially able to transform absorbed 3ADON and 15ADON into DON. An *in vitro* study has shown that isolated IEC are sensitive to 3/15ADON [60], proving that these derivatives could be directly absorbed by the IEC without the requirement of intestinal lipases or microbial lipases/esterases. The relative contribution of intestinal lipases, bacteria and IEC in the metabolism of acetyl-DON may depend of the animal species. In monogastric animals, the significance of the transformation of 3/15ADON by colonic bacteria is limited due to their high absorption by IEC [60], suggesting a major role of intestinal lipases and/or epithelial CES in that case (Figure 5). To date, direct evidence of the transformation by intestinal CES of 3/15ADON in DON are unfortunately missing. In ruminants and poultry, bacterial de-acetylation of 3/15ADON could happen prior the small intestine and may theoretically impact their bioavailability.

Little is known regarding the intestinal absorption and bacterial metabolism of the more polar metabolites of DON, *i.e*., D3G, D3GA and D15GA. On the basis of the hypothesis that DON enters IEC by lipid diffusion, such polar metabolites should have lower intestinal absorption efficiency compared to the native toxin. Similarly, one could suppose that addition of glucoside or glucuronide moiety would impact the interaction of DON with its membrane transporter, affecting their cell entry through this mechanism (see part 2.2.). No data exists regarding the intestinal absorption of D3/15GA, but the lack of toxicity of these metabolites suggests that their oral bioavailability is certainly low to nil [61]. Similarly, nothing is known regarding the bacterial metabolism of D3/15GA. One could suspect that bacterial beta-glucuronidases would certainly transform them into DON with or without consequences, depending if the transformation occurs prior to or after the small intestine. As with D3/15GA, D3G is unable to cause toxicity [62]. Data have, however, shown that intestinal bacteria are able to transform D3G into DON through the hydrolysis of its glucoside moiety [18,19,53,63,64]. Interestingly, the bacterial activity leading to the transformation of D3G in DON is spontaneously present in the feces and does not seem to require its induction as observed for the transformation of DON in DOM-1 [53]. In monogastric animals, the transformation of D3G into DON after the small intestine does not allow the absorption of the released DON since most of the toxin remains in the feces, suggesting that D3G is not hazardous at least for these animals due to its limited intestinal absorption (Figure 5) [64]. Again, the situation may be totally different in ruminants and poultry where the transformation of D3G in DON would take place before the small intestine, potentially allowing the absorption of the released toxin.

### 2.4. Metabolism and Excretion of DON and Its Derivatives by the Animals

The ingestion of native DON and its derivatives leads to the presence of a native toxin in the body of intoxicated animals. As with many xenobiotics, DON is then subject to detoxification and excretion (Figure 5, Figure 6).

Transport studies using Caco-2 cells have demonstrated that human IEC have the ability to apically excrete DON [21]. Whereas the apical (AP) to basolateral (BL) transport of DON by human IEC is insensitive to transporter’s inhibitors, its BL to AP excretion is sensitive to P-glycoprotein inhibitors, particularly inhibitors of the multidrug resistance-associated protein (MRP-2) transporter [21]. In addition to reduce the net absorption of ingested DON by IEC of the small intestine, net excretion by IEC of the colon may account for the total excretion of DON (and possibly D3/15GA) by the body. Detoxification of ingested xenobiotics generally takes place in the IEC, the liver and the kidneys. Detoxification of DON certainly starts in IEC, directly after its intestinal absorption. Although the detoxification metabolite DOM-1 is present in the blood of animals orally intoxicated with DON, as mentioned above, the transformation of DON in DOM-1 is not related to animal detoxification as it occurs in the intestinal lumen and corresponds to bacterial detoxification followed by the intestinal absorption of DOM-1 [45,65]. Body detoxification of DON mostly involves the formation of glucuronide metabolites (mainly D3GA and D15GA) by UDP-glucuronosyltransferases. Such metabolites are less toxic than the parental toxin due to their lower log*D* value (Figure 2) making them less efficient at crossing the cell membrane and/or at binding to ribosomes [61]. The amount of glucuronide-DON formed greatly differs, depending of the animal species used. Thus, in sheep, glucuronide metabolites correspond to 75% of the systemic DON [46], whereas in pigs, the percentage of glucuronide metabolites varies from 5% to 58%, depending if the animals were exposed to DON or 3ADON, respectively [33,52]. This suggests that in addition to the animal species used, the form of the ingested toxin, either native or conjugated, also impacts its detoxification, at least in pigs. The precise site of the formation of the glucuronide-DON is not characterized at present, intestinal, liver and kidney cells being theoretically able to form glucuronide metabolites. Liver microsomes extracted from animals and humans have been shown to be able to transform DON in glucuronide-DON, mainly D3GA and D15GA [66,67]. Experiments conducted with intestinal or renal microsomes are not available to confirm that these tissues could also detoxify DON. However, the fact that in sheep the amount of glucuronide-DON formed is higher after oral exposure to DON (75%) than after intravenous injection (21%) suggests that IEC are responsible for a large part of the formation of such detoxification products in case of oral intoxication [46].

Regarding the excretion of DON, it seems that most of the toxin is excreted in the urine as glucuronide-DON, glucuronide-DOM-1, DON and DOM-1 (Figure 5, Figure 6). A study in humans has shown that 91% of the DON excreted in urine is glucuronide-DON, D15GA being predominantly found [68,69]. In pigs orally intoxicated by DON, 68% of the toxin is excreted in the urine as unchanged DON and glucuronide-DON, the remaining being mostly eliminated in feces (20%) as DOM-1 and DON (80% and 20% of total DON in feces, respectively) [32,34]. Similarly, oral exposure of pigs to 3ADON leads to the elimination of the toxin mainly in urine (up to 80%) as DON and glucuronide-DON with only low amounts of toxin (2%) being present in the feces as DON and DOM-1 (48% and 52%, respectively) [52]. As mentioned previously, intoxication of rats with D3G does not require body detoxification since most of the D3G is not absorbed; 3.7% of the toxin is eliminated in the urine as DON, glucuronide-DON and D3G, and all the rest is eliminated in feces as DON and DOM-1 [64]. Mechanism(s) of excretion of DON/DOM-1 and glucuronide-DON/DOM-1 are unknown at present and could involve both glomerular filtration of the metabolites present in the blood and their excretion through *P*-glycoproteins expressed by intestinal, renal or hepatic epithelial cells, as demonstrated for DON in IEC [21].

Overall, excretion of DON and its detoxification metabolites is quite efficient, half of plasmatic DON being eliminated after 6 h in pigs and in sheep [34,46]. The fast elimination of DON suggests a low binding of DON and its metabolites to serum albumin, at least in animals. Accordingly, the *in vitro* toxicity of DON is not modified by the presence of bovine serum albumin (BSA) in the medium, contrary to the toxicity of ochratoxin A, another mycotoxin with a strong affinity for serum albumin and a longer plasmatic half-life (up to 840 h) [70,71]. It has to be noted that a recent study described the interaction of DON with human serum albumin [72], indicating that in humans, DON could possibly have a longer plasmatic half-life.

### 2.5. Transport of DON through the Blood-Brain Barrier (BBB)

As described in Section 3.4., DON is able to cause alterations of the brain functions. Although part of these alterations could be attributed to peripheral effects, data suggest that neurologic effects of DON rely in part to the direct action of DON on brain cells. This requires the crossing of the blood-brain barrier (BBB) by the toxin. The BBB is formed by the close apposition of endothelial and glial cells, forming a selective barrier controlling the passage of molecules from the plasma to the cerebro-spinal fluid (CSF) [73]. *In vivo* studies have shown that DON crosses the BBB in various animal models. DON transport across the BBB occurs rapidly, native toxins being detected in the brain of animals within a few minutes (2 to 60 min, depending of the animal species) after exposure [74]. The ability of DON to cross the BBB depends on the animal species. In pigs, 25% to 30% of the plasmatic DON is found in the CSF, the toxin having a CSF half-life similar to its plasmatic one and being still detectable in the CSF 20 h after the intoxication [74]. In mice, the BBB crossing of DON is lower, the concentration of DON in the brain corresponding to 10% of the plasmatic concentration [75]. Finally, in sheep, only 5% of the plasmatic DON crosses the BBB [74]. Transport of DON across the BBB of other animal species and humans has not yet been evaluated, though it would not be surprising that some animal species may have higher or lower BBB permeability to DON. The best example of variation of BBB permeability for a specific molecule between animal species comes from another family of mycotoxins: the fumonisins. Indeed, BBB permeability to these toxins ranges from low/nil (for mice) to high (for horses) [76,77]. Importantly, it was demonstrated that perturbations of the BBB permeability, caused by LPS-induced neuro-inflammation, increase the brain accumulation of fumonisins in mice [78]. At present, no studies have looked at the effects of neuro-inflammation and perturbations of the BBB on the brain accumulation of DON. Similarly, nothing is known regarding the mechanism(s) responsible for the transport (absorption and excretion) of DON across the BBB. Regarding the ability of DON derivatives to enter the brain, only native toxin is found in the CSF of intoxicated animals, suggesting that neither DOM-1, nor D3/15GA are able to cross the BBB [74].

## 3. Pathophysiological Effects of DON

### 3.1. Cellular Effects of DON

As with other trichothecenes, DON is able to cause cellular effects through its ability to target ribosomes and to cause ribotoxic stress [4,5,6,7,8]. Trichothecenes have all in common an epoxide group at position 12–13 critical for their action on ribosomes, explaining why the de-epoxide diene metabolite DOM-1 is non-toxic [2,3,4,5].

**Figure 7:**
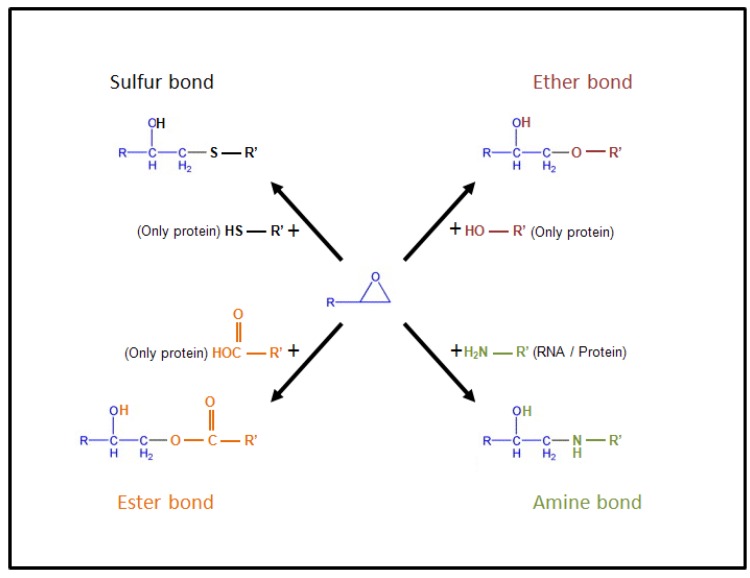
Chemical reactivity of the epoxide moiety. Epoxide moiety of DON could theoretically react with nucleophile functions present on the puric/pyrimidic bases of the nucleotides forming nucleic acid (DNA and RNA) such as amine group and/or on the side chain of amino acids forming the proteins such as: amine, hydroxyl, carboxyl and thiol groups.

Binding of DON to the ribosomes could occur through the reaction of the epoxide moiety of DON with the nucleotides forming ribosomal RNA (rRNA) [6,7,8]. Nucleotides contain amine groups potentially able to react with epoxide (Figure 7) [79]. At present, nothing is known regarding the precise nature of the chemical reaction(s) allowing DON to bind to rRNA. Surprisingly, no report has been made of the interaction of DON with other nucleotide-containing molecules, such as mRNA or DNA. Aflatoxins (AFL), another family of mycotoxins with an epoxide function after their metaboliation by CYP450, selectively bind to guanine and cytosine residues present both in DNA and RNA [80]. This suggests—if the absence of reaction between DNA/mRNA and DON is confirmed—either that nucleotides from rRNA have a particular spatial organization allowing their specific interaction with DON, or that the real target of DON in rRNA is not nucleotides.

**Figure 8:**
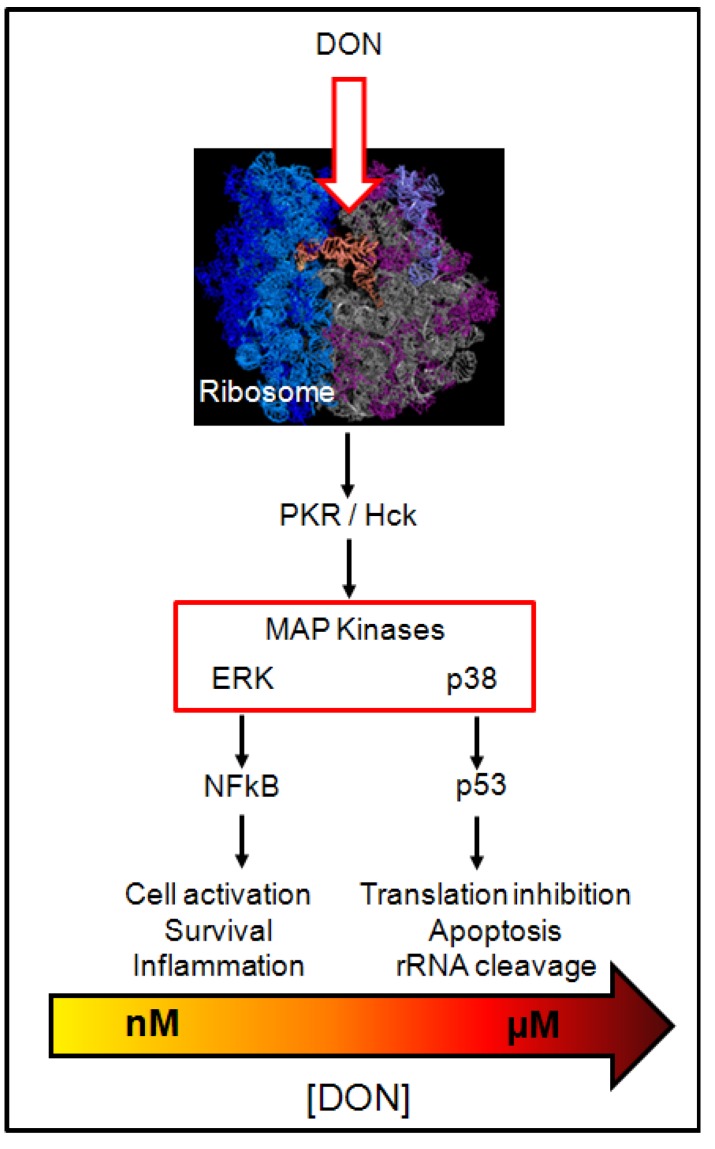
Cell effects of DON. Effects of DON on cell signal pathways in macrophages. Top image shows the organization of eukaryotic ribosome. The small subunit (40S) on the left contains an RNA molecule (cyan) and 20 proteins (dark blue); the large subunit (60S) on the right contains two RNA molecules (grey and slate) and more than 30 proteins (magenta). The image also shows a transfer RNA (orange) bound to the active site of the ribosome.

Proteins possess amine, carboxyl, thiol and hydroxyl groups all potentially able to react with the epoxide function of DON (Figure 7), as demonstrated for AFL that forms adduct with the amine function of the lateral chain of lysine residues in serum albumin [81]. Thus, one could imagine that the binding of DON to rRNA takes place through its interaction with rRNA-associated protein(s). However, in that case again, it is not clear why DON only targets such rRNA-associated protein(s) and not other cellular proteins. One could suppose that only rRNA possesses both the correct spatial organization and chemical functions, present either on nucleotides or rRNA-associated proteins, allowing their selective interaction with DON. What we know is that the binding of DON to rRNA causes their cleavage and the activation of various cellular signaling pathways affecting cell functions and potentially leading to cell apoptosis (Figure 8) [6,7,8,82,83]. Signal pathways activated by DON correspond to the one generally activated by ribotoxins and ribotoxic stress, including: (i) two rRNA associated protein kinases, *i.e*., the double-stranded RNA (dsRNA)-activated protein kinase (PKR) and the hematopoietic cell kinase (Hck); and (ii) the MAP kinases (p38, ERK1/2, JNK), affecting the expression of proteins involved in the innate immunity (through NFκB activation) and apoptosis (through p53) [6,7,8].

Initially, it was proposed that the binding of DON to rRNA cause their cleavage that in turn activate PKR and Hck, leading to the downstream activation of MAP kinases, NFκB and apoptosis pathways [6]. But recent elegant work from Pestka’s group has shown that, in fact, apoptosis activation is not the consequence but is rather the cause of the rRNA cleavage through the activation of caspases and RNases [82,83]. In accordance with this hypothesis, the authors showed that signaling pathway activation occurs in minutes after exposure to low or high doses of DON, whereas rRNA cleavage appears only after hours of exposure to high doses. In addition, authors demonstrated that inhibitors of signal pathways and apoptosis inhibit rRNA cleavage caused by DON, thus definitively proving that rRNA cleavage is the consequence of cell signaling induced by DON and not the opposite.

The actual hypothesis regarding DON effects is that after its cell entry, DON binds to rRNA through the interaction of its epoxide moiety with functional group(s) present on the nucleotides and/or rRNA-associated proteins (such as PKR and Hck) leading to the rapid activation of the rRNA-associated protein kinases PKR and Hck which then activate MAP kinases, the type of MAP kinases activated being different depending of the doses of DON used. Thus, in macrophages, low doses (nM) activate preferentially ERK, causing cell survival and gene expression, whereas high doses (µM) activate p38 leading to apoptosis, rRNA cleavage and protein synthesis inhibition (Figure 8).

At low doses, DON has been showed to regulate the expression of various genes involved in the innate immunity and the inflammatory reactions through selective transcription, mRNA stabilization and translational regulation [7,8,82,83]. In addition to PKR, Hck, MAP kinases and NFκB other proteins participate in the transcriptional/translational effects of DON, including the HuR/Elav-like RNA binding protein 1, the CCAAT/enhancer-binding protein (CHOP) homologous protein, the peroxisome proliferator-activated receptor γ (PPARγ), the early growth response gene 1 (EGR-1), the activating transcription factor 3 (ATF3), the histone methylase, and GRP78/BiP [84,85,86,87,88,89]. It will not be surprising that additional signaling proteins participate in DON effects. Accordingly, a recent study from Pestka’s group showed that DON affects the phosphorylation of 188 proteins, including proteins involved in transcription, epigenetic modulation, cell cycle, RNA processing, translation, ribosome biogenesis, cell differentiation and cytoskeleton organization [90].

Few studies have looked at the cell effects of DON derivatives. It has been known for decades that the loss of the epoxide moiety leads to the absence of cell effects of DOM-1 due to its inability to bind to ribosome independently of its cell entry [18]. More polar derivatives of DON (D3/15GA and D3G) are also non-toxic due either to their inability to cross the cell membrane and/or to bind to ribosomes, the relative participation of each event being unknown at present [61,62]. Toxicity of the less polar derivative (3/15ADON) depends of the organ and animal species, differences in their ability to enter the cells and/or to bind to ribosomes compared to DON possibly being involved. Using lymphocytes, 3ADON and 15ADON were found less toxic than DON [91,92,93]. Conversely, using pig IEC and intestinal explants, Oswald’s group ranked the toxicity of DON and its acetylated derivatives as follows: 3ADON < DON < 15ADON [60]. *In vivo* experiments on mice confirmed the higher intestinal toxicity of 15ADON compared to DON in case of ingestion but not after i.p. injection, suggesting a particular sensitivity of the intestinal epithelium to acetyl-DON [94]. Based on the fact that ribosomes are theoretically the same in all cells, the difference of sensitivity of lymphocytes and IEC to acetyl-DON suggests that acetylation may affect the cell entry of DON derivatives, IEC being more efficient at transporting acetyl-DON than lymphocytes. Another attractive hypothesis would be that DON and 3/15ADON have a similar ability to enter the cells, but that only DON, and not 3/15ADON, binds to ribosomes. In that case, the higher sensitivity of IEC compared to lymphocytes could rely on the higher ability of IEC to transform 3/15ADON into DON through CES activity.

Overall, according to their ability to enter the cells, only DON and 3/15ADON have been shown to affect the functions of intestinal, immune and brain cells; DON effects on these systems are interconnected as described below.

### 3.2. Impacts of DON on the Intestinal Functions

**Figure 9:**
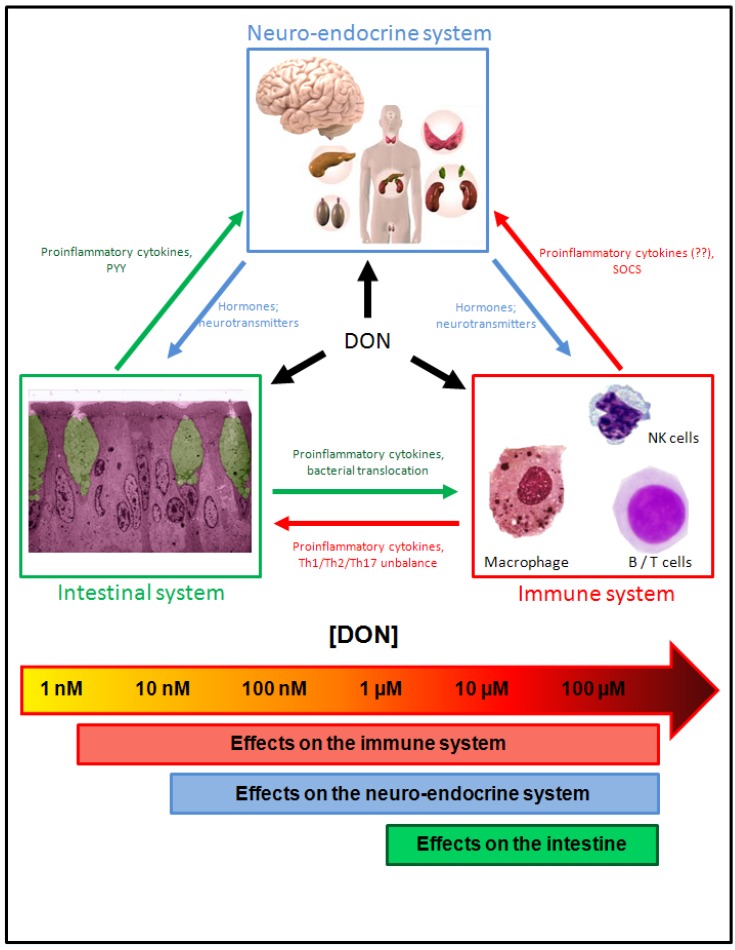
Effects of DON on the intestinal, immune and neuro-endocrine systems. Effects of DON on the intestinal, immune and neuro-endocrine systems are explained in the text. Doses at which the effects occur are schematically indicated at the bottom of the figure. It appears that the order of sensitivity of the systems is as follow: immune > neuro-endocrine > intestinal (Intestinal microscopy image courtesy of Cendrine Nicoletti).

Intestinal epithelial cells (IEC) are the first target of DON in case of natural exposure through ingestion of contaminated food. Whereas only IEC of the small intestine are exposed apically to ingested DON, IEC of the small intestine and colon are potentially exposed basolaterally to systemic DON that has crossed the intestinal wall to reach the blood compartment. Numerous studies have demonstrated that DON impacts IEC functions (Figure 9 and Table 1) (for review: [14,16,95]).

DON alters the proliferation and viability of animal and human IEC. In human IEC, the inhibition of the cell proliferation is observed at low doses (IC_50_ = 1–5 µM), cytotoxic effects being observed at higher doses (30–40 µM) [39,96]. Similarly, high doses of DON (IC_50_ = 10–50 µM) cause cell toxicity and apoptosis in rat and pig IEC [97,98,99,100]. Importantly, studies conducted on pig IEC have shown that the status of the cells (undifferentiated *versus* differentiated) and the site of DON exposure (apical or basolateral) affect its toxicity, DON being more toxic to undifferentiated IEC and when added basolaterally [97,98,99]. Whereas the higher sensitivity of undifferentiated cells compared to differentiated cells (10-times more sensitive) may be explained by differences in their cell cycle, no formal reasons explain the higher susceptibility of cells exposed basolaterally compared to apically (4-times difference), especially on the basis of the supposed passive diffusion entry of DON in the cells. No studies have been conducted to see if IEC absorb/accumulate more DON when the toxin is added basolaterally, explaining such a difference. Experiments need to be performed to understand how the site of exposure affects so much the effect of DON on IEC both in terms of toxicity and gene expression [97,98,101].

*In vitro* and *in vivo* experiments have also shown that DON inhibits the intestinal absorption of nutrients (at least glucose and amino acids) by human [39] and animal IEC [37,102,103,104]. The sodium-glucose dependent transporter (SGLT-1) activity is particularly sensitive to DON inhibition with an IC_50_ of 10 µM [39]. In addition to nutritional consequences, inhibition of SGLT-1 could explain the diarrhea associated with the ingestion of DON, since this transporter is responsible for the daily absorption of 5 L of water by the gut [105]. How DON causes inhibition of SGLT1 and other nutrient transporters is unknown at present, this inhibition being possibly related either to non-specific effects such as protein synthesis inhibition or ATP depletion, or to specific modulation of the expression/membrane targeting/activity of the transporters. According to the second hypothesis, activation of MAP kinases in IEC by proinflammatory signals causes the inhibition of the activity of membrane inserted SGLT-1 without affecting its expression [106,107].

Based on the ability of DON to activate MAP kinases in IEC, it would not be surprising that DON inhibits glucose absorption through such a mechanism. Finally, although no data are available to support such a hypothesis, one could speculate that D3G could act as a competitive inhibitor of SGLT-1 through its glucoside moiety.

**Table 1:** Risk evaluation of the exposure to DON for humans. Risks associated to DON exposure in humans were evaluated using the doses required to cause physiological alterations and PMTDI/higher exposure of the human population. Intestinal, blood and CSF concentrations of DON were calculated on the basis of a human adult weighting 70 kg, having a small intestinal volume of 1 L, a blood volume of 5 L and assuming that humans behave like pigs regarding blood and CSF concentration of DON [14,74]. The physiological alterations occurring at doses of DON with a safety factor inferior to 30 compared to the PMTDI/highest exposure-related concentrations are indicated in red.

Organ/system affected	Effect	Doses required	Times the PMTDI-related concentrations	Times the highest dose-related concentrations
***INTESTINE***	Inhibition of the cell proliferation	1–5 µM	**4.8–23**	**2–10**
	Increase in β-Defensin expression	2 µM	**10**	**3.9**
	Decrease in nutrient absorption	10 µM	48	**19**
PMTDI-related intestinal concentration = 210 nM	Decrease in mucin expression	10 µM	48	**19**
	Increase in intestinal pathogenic Th17	10 µM	48	**19**
	Increase in bacterial translocation	10 µM	48	**19**
	Increase in IL-8 secretion	1–20 µM	48–95	**2** –39
	Modification of the microbiota	20 µM	95	39
Highest dose-related intestinal concentration = 504 nM	Increase in intestinal permeability	10–50 µM	48–245	19–99
	Cytotoxicity	>30 µM	142	59
	Decrease in IL-8 secretion	>30 µM	142	**59**
	Increase in IgA secretion	500 µM	2 380	992
***IMMUNE SYSTEM***	Increase in lymphocyte proliferation	1–30 nM	**0.6–20**	**0.3–8**
	Activation of macrophages	1–100 nM	**0.6**–66	**0.3–28**
PMTDI-related blood concentration = 1.5 nM	Increase in proinflammatory cytokines	0.1–1 µM	66–666	**28** –277
	Decrease in lymphocyte proliferation	>100 nM	>66	>28
	Inhibition of NK cells	>150 nM	>100	>41
Highest dose-related blood concentration = 3.6 nM	Apoptosis of macrophages	>300 nM	>200	>83
	Apoptosis of lymphocytes	10 µM	6666	2777
***ENDOCRINE SYSTEM***	Steroid perturbations	0.3–3 µM	200–2 000	83–833
	Increase in insulin secretion	1.44 µM	960	400
	Decrease in IGF-1/IGFALS	1.8 µM	1200	500
PMTDI-related blood concentration = 1.5 nM Highest dose-related blood concentration = 3.6 nM	Increase in secretion of PYY	7 µM	4666	1944
***BRAIN***	Feed refusal	1.5–75 nM	**3** –166	**1.4** –69
	Activation of microglia	10–100 nM	**22** –222	**9** –92
PMTDI-related brain concentration = 0.45 nM	Inhibition of glutamate uptake	50 nM	111	46
	Cell death/Inhibition of microglia	>300 nM	>666	>277
	Vomiting	1.2 µM	2666	1111
	Direct neuroinflammation	1.5 µM	3333	1388
Highest dose-related brain concentration = 1.08 nM	Cell death of astrocytes	31 µM	68,888	28,703

In addition to directly affecting the activity of nutrient transporters, DON also affects the permeability of the intestinal epithelium through modulation of the tight junction complexes (IC_50_ = 10 to 50 µM) [20,39,40,60,97,98,100,108]. Studies have demonstrated that activation of MAP kinases (particularly ERK) by DON affects the expression and cellular localization of proteins forming or being associated with the tight junctions such as claudins, ZO-1, resulting in an increase in the paracellular permeability of the intestine [20,60,97,98,108]. Acetylated DON derivatives are also able to affect the tight junctions through activation of the MAP kinases pathway, a direct correlation existing between their ability to activate MAP kinases and to open tight junctions, with the following order 3ADON < DON < 15ADON [60]. In addition to affecting nutrient absorption and causing intestinal inflammation [14,40], the increase in paracellular intestinal permeability may explain why animals chronically exposed to the toxin have higher DON oral bioavailability [32]. Interestingly, others in addition to us reported that incubation of IEC with doses of DON not able to affect the tight junctions causes a transcellular bacterial translocation across the intestinal epithelium suggesting a possible role of DON as risk factor for inflammatory bowel diseases (IBD) and intestinal bacterial infections [14,40,99,109]. In addition to opening tight junctions and promoting bacterial translocation, DON also modifies the production of the intestinal mucus. Thus, *in vivo* studies with pigs have shown that ingestion of DON causes a decrease in the number of goblet cells and in the production of mucus [110,111], potentially explaining the observed perturbations of the microbiota in pigs exposed to DON [14,112]. Accordingly, we have preliminary *in vitro* data showing that DON modifies the expression/production of mucins by human cells exposed to 10 µM of toxin (personal communication).

Finally, innate immunity related to IEC is also affected by DON both directly (through the activation of signal pathways by the toxin) and indirectly (through the crossing of luminal bacterial antigens caused by the bacterial translocation, mucus alteration and the opening of the tight junctions) [14,40]. Thus, DON (1 to 20 µM) affects the expression of proteins involved in the epithelial innate immunity, including COX-2 and β-defensins [113,114,115]. Similarly, numerous studies using animal and human cells have demonstrated that DON stimulates the expression and secretion of interleukin-8 (IL-8), a chemoattractant cytokine causing the recruitment/activation of circulating immune cells and thus potentially participating indirectly in the central effects of DON in terms of feed refusal and emesis. Induction of the intestinal inflammation by DON takes place through the activation of PKR/Hck/MAP kinases/NFκB pathways [40,86,89,99,114,116,117]. Study with human IEC has shown that DON has a biphasic effect on the secretion of IL-8, low doses of toxin (1 to 25 µM, non-cytotoxic) causing a massive increase in the secretion of IL-8, whereas higher doses (50 to 100 µM, cytotoxic) inhibit it [40]. Similarly, as described for immune cells, low doses (10–20 µM) of DON potentate the effects of pro-inflammatory molecules such as cytokines or bacteria components (flagella, LPS) on intestinal IL-8 secretion, whereas higher doses of DON inhibit it [40,116]. Taken together, such a biphasic effect explains why DON acts: (i) as a proinflammatory toxin leading to intestinal inflammation at low doses; and (ii) as an inhibitor of the intestinal immunity leading to higher susceptibility of animals to intestinal infections at higher doses [14,40,118,119].

Taken together, the opening of tight junctions, the increase in the bacterial translocation, and the decrease in the mucus production caused by DON may promote the passage across the intestinal epithelium of xenobiotics (pharmaceutics, pesticides, others mycotoxins), harmful molecules (prion, bacterial toxins, alimentary allergens) and pathogenic micro-organisms (bacteria, fungi, viruses) present in food and water.

As detailed below, in addition to local effects, the alterations by DON of the intestinal functions, including epithelial innate immunity, may have consequences on the systemic immunity (part 3.3.) and on the brain functions (part 3.4.).

### 3.3. Impacts of DON on the Immune Functions

The second organ system targeted by DON once the toxin has crossed the intestinal epithelium is the immune system. *In vivo* and *in vitro* studies have shown that immune cells (including macrophages, B and T lymphocytes and natural killer (NK) cells) are very sensitive to DON and its toxic derivatives (3/15ADON), exposure to the toxin leading either to immunostimulatory/inflammatory or immunosuppressive effects depending of the dose, as demonstrated with IEC (Figure 9 and Table 1) [5,6,7,8,12,93,120,121].

Due to their ability to phagocytose pathogens, to present antigens and to secrete cytokines regulating B/T cells functions, monocytes/macrophages are critical in the immune system as they link together the innate and the acquired immune responses [122]. Macrophages are highly sensitive to DON exposure. Stimulation of macrophages with low doses of DON (nM range) causes their activation, the secretion of inflammatory cytokines such as IL-1β, IL-2, IL-4, IL-5, IL-6 and TNFα and the expression of intracellular proteins involved in the innate immunity such as COX-2 and iNOS through the selective activation of ERK, NFκB and activator protein-1 (AP-1) [5,6,7,8,123,124,125]. In addition to its direct stimulatory effect, DON at low doses also potentates the stimulatory effects of cytokines/bacterial components on macrophages [124,125]. In parallel to macrophage activation, low doses of DON also affect their ability to phagocytose and to kill bacteria, leading either to a decrease or an increase in the phagocytosis depending of the type of bacteria used in the assay [109,126]. As shown with IEC, higher doses of DON (µM range) possess suppressive effects on macrophage activations (cytokine secretion, phagocytosis, bacterial killing) and induce their apoptosis [124,125,127] such deleterious effects certainly contributing to the observed increase in the susceptibility to infection of animals exposed to DON [119,127,128]. As mentioned in part 3.1., it has to be noted that both macrophage activation and apoptosis induced by DON depend on the type of MAP kinases activated, *i.e*., ERK for the survival/activation signal and p38 for the inhibition/pro-apoptotic signal [6,7,8]. It is interesting to note that macrophages are the most sensitive cells regarding DON toxicity, such cells being 10 to 100-fold more sensitive compared to other cell types, including fibroblasts, lymphocytes, IEC or astrocytes. Hypotheses could only be formulated regarding such differences, the higher sensitivity of macrophages to DON toxicity relying either on: (i) a potential and unproved higher ability of DON to enter/accumulate in these cells; and/or (ii) on a specific activation of JAK/STAT pathway leading to apoptosis in these cells [129]. In addition to impacting the innate immunity, alterations of the macrophage functions by DON also affect the acquired immune response. Thus, the decrease in the phagocytosis/bacterial killing and cytokine production induced by DON may inhibit the ability of macrophages to play their role as antigen-presenting cells (APC) and to activate B and T cells. Accordingly, macrophage perturbation was proposed to play a role in the aberrant production of IgA by the B cells of the intestinal Peyer’s patches [130,131].

Independently of the alterations of the macrophages, DON also affects the proliferation and functions of lymphocytes, including B, T and NK cells.

Natural killer (NK) cells are effector lymphocytes of the innate immunity playing an important role in the immune surveillance against tumors and microbial infections [132]. Low doses of DON (150–300 nM) are able to inhibit the activity of human NK cells suggesting that DON exposure could indirectly favor the emergence of tumors through a decrease in the immune vigilance associated to NK cells, at least in humans [93].

DON also affects lymphocytes of the acquired immunity (B and T cells). At high doses (superior to 10 µM), DON causes the apoptosis of lymphocytes, leading to immuno-suppression, increased susceptibility to infection, reactivation of latent infections and decreased vaccine efficiency [6,7,8,119,128,133,134]. At lower doses, DON has a biphasic effect on the mitogen-induced proliferation of human and animal lymphocytes, 1 to 30 nM of toxin stimulating the proliferation, whereas 100 to 600 nM of DON suppress it [134,135]. At low doses (nM), DON also increases the expression of cytokines by lymphocytes, including IL-2, IL-4, IL-6, IL-8 and TNFα [136]. Alterations of the lymphocyte proliferation and of the secretion of particular cytokines may explain the imbalance in the Th1/Th17/Th2 immune responses observed after intoxication of the animals with DON. In mice, DON exposure results in a parallel suppression and stimulation of the systemic Th1 and Th2 immune responses, respectively [119]. Similarly, exposure of intestinal explants from pigs to DON at 10 µM causes a profound alteration of the intestinal Th17 immune response with a selective increase in the expression of genes associated to the pathogenic/inflammatory Th17 cells (*i.e*., IL-23A, IL-22, IL-21) without affecting the expression of the genes associated to the regulatory/protective Th17 cells (*i.e*., the anti-inflammatory cytokine IL-10 and TGF-β) [117]. Modification of the secretion of cytokines by T cells and macrophages located in the Peyer’s patches could also explain how DON modifies the production of antibodies by the B cells, the exposure to DON being characterized by an increase in the production of IgA and a parallel decrease in the production of IgM and IgG [130,131,134,137]. Importantly, part of the IgA produced after exposure to DON reacts with self-antigens and gut bacteria as observed in IBD [138]. Based on the ability of DON to cause intestinal and immune alterations mimicking the one found in IBD, we proposed in 2010 that DON could play a role in such diseases, our hypothesis being now defended by others and, more importantly, being confirmed by the recent work conducted on pigs by Oswald’s group showing the activation of intestinal pathogenic Th17 at 10 µM of DON [14,117].

The effects of DON derivatives on immune cells have been studied. As observed with other cell systems, DOM-1 and glucuronide-DON have been found non-toxic to immune cells [61,139], no studies having tested the effect of D3G. Regarding acetyl-DON derivatives, it has been shown that 3ADON and 15ADON are less toxic than DON to human and mouse lymphocytes [91,92,93,120], difference compared to DON in their ability to enter the cells and/or to bind to ribosomes potentially explaining it (see part 3.1.).

In addition to affecting the immunity, alterations of the immune cells by DON could affect the intestinal and the brain functions. Indeed, local activation of intestinal immune cells by DON could reinforce the direct proinflammatory effect of DON on IEC through a vicious circle in which IEC and immune cell-mediated inflammations potentate each other as described in IBD [14]. In addition, intestinal and systemic production of cytokines could affect the endocrine system and the brain functions and thus participate in the growth retardation, feed refusal and emesis caused by DON as explained below.

### 3.4. Impacts of DON on the Brain and Endocrine Functions

Studies have demonstrated that DON affects the nervous and the endocrine systems (Figure 9 and Table 1).

Regarding the endocrine perturbations, it was shown that DON (at 0.3–3 µM) modifies the gene expression, viability and synthesis/secretion of steroid hormones by human adrenocortical cells, causing an increase in the secretion of progesterone and a parallel decrease in the production of testosterone, estradiol and cortisol [140]. Stimulatory effect of DON on the secretion of progesterone was furthermore confirmed in animals, such endocrine perturbation potentially leading to reproductive toxicity [141,142]. Systemic inflammation induced by nanomolar doses of DON also causes the production of suppressors of cytokine signaling (SOCS) able to inhibit the induction by the growth hormone of the hepatic secretion of IGF-1 and IGF acid labile subunit (IGFALS) eventually resulting in growth retardation [143,144]. Finally, DON increases the secretion of insulin and of the gut satiety hormone peptide YY (PYY), two hormones with anorexic action [145,146]. Importantly, antagonist of the PYY receptor partially prevents the anorexigenic effect of DON, showing that PYY plays a role in the anorexia induced by DON [146].

In addition to endocrine perturbations, DON causes perturbations of brain cells. As mentioned in Section 2.5., part of the plasmatic DON is able to cross the BBB to directly act on neurons and glial cells forming the brain [74,75]. An *in vitro* study conducted on brain cells isolated from newborn rats has shown that DON affects the viability and functions of astrocytes and microglial cells [147]. The sensitivity of astrocytes to DON toxicity is similar to the one observed with epithelial cells or lymphocytes (IC_50_ of 31 µM on the cell viability). Microglial cells, in accordance with their origin (monocytes) are much more sensitive to DON toxicity with an IC_50_ of 259 nM on cell survival (more than 100-fold difference compared to astrocytes). Whether or not the higher sensitivity of microglia to DON toxicity relies on JAK/STAT pathway activation as observed for monocytes/macrophages [129] remains to be determined. In addition to affect their viability, DON is also able to modify the functions of glial cells. DON has a biphasic effect on the microglia-associated neuro-inflammation [147]. At doses inferior or equal to 100 nM, DON potentates the neuro-inflammation caused by LPS in terms of iNOS induction and TNF-α secretion. Conversely, at doses superior to 300 nM, DON dose-dependently inhibits the neuro-inflammation induced by LPS certainly through a general cytotoxic effect of DON on microglia [147]. We also found that DON, at doses not causing toxicity to astrocytes, inhibits their ability to reabsorb the excitatory neurotransmitter glutamate through EAAT1/2 transporters (IC_50_ = 50 nM, total inhibition at 1 µM) [147]. Surprisingly and contrarily to what we found with another mycotoxin, ochratoxin A [148], such inhibition is associated to a massive increase in the membrane expression of EAAT1/2 through an unidentified mechanism. Inhibition induced by DON of the glutamate uptake by astrocytes may have major consequences since this activity prevents neuronal damage caused by high excitotoxic extracellular glutamate concentrations [149] and that perturbation of glutamate clearance by astrocytes could also contribute to brain tumor progression [150], pain hypersensitivity [151] and to alterations in learning and memory consolidation [152]. Although very interesting, these *in vitro* data showing the perturbation of glial cells by DON now need to be confirmed by *in vivo* studies.

*In vivo* studies have shown that DON affects the activity of brain neurons, particularly in relation to anorexia and emesis; exposure of pigs to 10–75 or >150 µg of DON/kg BW (body weight)/day causing partial/total feed refusal or vomiting, respectively (for review: [153]). Importantly, higher doses of DON are required in mice, *i.e*., 0.5 to 5 mg/kg of BW causing anorexia, suggesting that pigs are more sensitive to brain effects than mice [153]. This could be related to the higher ability of DON to cross the BBB in pigs compared to mice (30% *versus* 10% of the plasmatic DON reaching the CSF in pigs and mice, respectively [74,75]) and/or to a higher sensitivity of pigs to emetic/anorexigenic stimuli, the important question being whether or not humans are closer to pigs or mice regarding the brain effects of DON. Regarding the mechanism involved in feed behavior effects of DON, it was first shown that emesis and anorexia induced by DON rely on central serotoninergic activities, as demonstrated for other emetic molecules [154,155,156]. An *in vivo* study conducted on rats next identified a role of neurons from the area postrema in the DON-induced conditioned taste aversion [157]. More recently, *in vivo* studies proved that oral exposure to DON at 1 mg/kg of BW and at 6 to 25 mg/kg of BW in pigs and mice, respectively, activates central anorexigenic neurocircuitries, including POMC and nesfatin-1 neurons present in specific area of the brain controlling the food intake and the vomiting [158,159,160]. Furthermore, it was demonstrated that, in addition to systemic/peripheral inflammation, DON also causes a central neuro-inflammation with an increased expression of proinflammatory molecules in the brain, including IL-1β and TNF-α and the anorexigenic prostaglandin PGE2 synthesized by mPGES-1 [159]. Although it was initially proposed that central and/or peripheral inflammation may cause the DON-induced anorexia as observed with LPS [161], *in vivo* data do not support such a hypothesis. Indeed, inhibition of the TNF-α signaling does not affect DON-induced anorexia [162]. Similarly, the section of the vagus nerve known to prevent the anorexigenic effect of peripheral inflammation induced by LPS does not affect DON-induced brain activation [158]. Finally, DON still causes anorexia in mPGES-1 knock-out mice that are resistant to anorexia induced by LPS, showing that peripheral and central inflammations caused by DON are not involved in the DON-induced anorexia and that, although LPS and DON activate a similar brain area, they use different mechanisms to do so [159]. At present, the exact mechanism involved in DON-induced anorexia is still a mystery. One could speculate, based on an antagonist study, that the intestinal secretion of PYY induced by DON is totally responsible for its anorexigenic action [146]. However, the fact that the direct injection of DON in the CSF leads to activation of the anorexigenic neurons and to anorexia rule out such a hypothesis. Accordingly, although peripheral secretion of PYY could play a role, DON-induced anorexia certainly also depends on the central effect of the toxin independently of its neuro-inflammatory effect [159]. We could propose that DON either activates neurons directly involved in feed refusal and/or affects glial cells regulating anorexigenic neuronal circuitries [163]. Future studies should help confirm such a hypothesis.

Not a lot of studies have looked at the brain effects of DON derivatives. 3ADON and 15ADON possess similar anorexic effects compared to DON, potentially in accordance with the fast and efficient conversion of such derivatives in DON when they enter the body [164]. The absence of DOM-1 and D3/15GA in the CSF of intoxicated animals suggests that such metabolites are not able to cross the BBB or to enter the brain [74].

## 4. Conclusions: Global View of the Effects of DON and Risk Assessment for Humans Exposed to DON

*In vivo* and *in vitro* studies have demonstrated that DON is able to alter the functions of the gut, the immune system, the endocrine system and the brain, modifications of each system happening at specific doses of DON and potentially affecting the functions of the others (Figure 9 and Table 1). DON-induced perturbations of the intestinal functions and of the intestinal immunity are observed at micromolar doses. Although the intestine is thus the less sensitive organ system regarding DON toxicity, we have to remember that the intestine is also the organ system exposed to the higher doses of DON, making DON-induced perturbation of the gut likely in case of ingestion of the toxin. In addition to affecting the gut functions, intestinal effects of DON also lead to alteration of the systemic immunity and of the endocrine/brain systems through the release of proinflammatory cytokines and of gut-associated hormones, such as the anorexigenic hormone PYY. Alterations of the immune system observed at nanomolar to micromolar concentrations of DON, in addition to affecting the immunity, may impact the intestinal and the neuro-endocrine functions through a vicious cycle, as observed in IBD or in the case of the peripheral inflammation caused by LPS [14,161]. Finally, perturbations of the neuro-endocrine system, in addition to causing modifications of the behavior including appetite, in turn affect the gut and the immune system functions through the release of neuro-endocrine mediators. Importantly, DON-induced inflammation of the intestine and brain could increase the permeability of the intestinal and blood-brain barriers and thus increase the crossing of these barriers by DON (and others toxins), ultimately affecting its bioavailability and its toxicity.

The use of highly innovative and promising methods based on the measurement of exposure biomarkers has shown that humans are significantly exposed to DON and its derivatives [165,166,167]. Although DON-induced perturbations have been demonstrated both *in vivo* and *in vitro*, one major question remains: are the doses causing such alterations realistic? To be as straightforward as possible, are the doses causing intestinal/immune/neuro-endocrine effects susceptible to be reached in humans exposed to food contaminated by DON? To address this question, we compare in Table 1: (i) the concentrations of DON potentially found in the intestinal lumen, the blood and the CSF, based on its provisional maximum tolerable daily intake (PMTDI) and the higher range of exposure in adult and children to DON obtained from the Joint FAO/WHO Expert Committee on Food Additives (JECFA) [14]; to (ii) the doses of DON required to cause alterations in the gut, the immune/endocrine system or the brain. Concentrations of DON in the intestinal lumen, the blood and the CSF have been estimated using the PMTDI/higher exposure of DON and assuming that: (i) a human adult has a body weight of 70 kg, a global small intestinal volume of 1 L (considering the net intake/secretion (around 9 L) and absorption (around 8 L) of water by the gut), and a blood volume of 5 L; and that (ii) toxicokinetics data obtained with pigs orally exposed to DON could be extrapolated to humans [14,74]. On the basis of a PMTDI of 1 µg/kg of BW per day for DON, toxin concentrations should be: 210, 1.5 and 0.45 nM in the intestinal lumen, the blood and the CSF, respectively. On the basis of the worldwide higher exposure in adult and children to DON obtained from the Joint FAO/WHO Expert Committee on Food Additives (JECFA) (0.78 to 2.4 µg/kg of BW per day), DON concentrations would reach maximal values of 504, 3.6 and 1.08 nM in the intestine, the blood and the CSF, respectively.

From the analysis of Table 1, it clearly appears that, as suggested by others and us [14,168], DON represents a risk to human health based on the presence of a low safety factor (inferior to 30) between the doses of DON affecting cell functions and the doses of DON susceptible to be present in relation to its actual PMTDI. The risk concerns mainly the intestinal and immune systems and the brain; DON effects on the endocrine system are being unlikely to be observed in humans exposed to DON at doses close to its PMTDI. Importantly, the risk could be even higher than supposed since the toxicokinetic profile (intestinal absorption, detoxification, excretion, BBB crossing) and/or the cellular effects of DON could be affected by factors not considered in our calculation. This includes: (i) the concomitant presence in food of others xenobiotics and toxins such as drugs, heavy metals, pesticides, bacterial/plant toxins or others mycotoxins [169]; and (ii) the exposure of particular populations to DON, including: vegans/macrobiotics, children and patients suffering from bacterial/viral infection, renal/hepatic diseases, IBD, compromised immunity, neurological disorders or cancers, these populations being at higher risk regarding DON effects [14,170].

Taken together, such observations should alert food agencies and potentially lead to the reevaluation of the actual PMTDI for DON, particularly as new DON metabolites have been found in plants and food products, including DON-oligoglycosides, DON-glutathione, DON-*S*-Cysteine, DON-*S*-Cysteinyl-glycine, DON-sulfonate. Such derivatives represent new “masked” toxins not yet considered in the total intake of DON and for which few or no data are available regarding their intestinal transformation/absorption and their cellular toxicity [139,171,172].
